# Task sharing for family planning services, Burkina Faso

**DOI:** 10.2471/BLT.19.230276

**Published:** 2019-09-25

**Authors:** Tieba Millogo, Séni Kouanda, Nguyen Toan Tran, Boezemwendé Kaboré, Namoudou Keita, Leopold Ouedraogo, Fatim Tall, James Kiarie, Nandita Thatte, Mario Festin, Asa Cuzin-Kihl

**Affiliations:** a Institut de Recherche en Sciences de la Santé, Ouagadougou, Burkina Faso.; b Australian Centre for Public and Population Health Research, University of Technology Sydney, Sydney, Australia.; c Family Planning Service, Health Ministry, Ouagadougou, Burkina Faso.; d West African Health Organization, Bobo-Dioulasso, Burkina Faso.; e WHO Regional Office for Africa, Brazzaville, Congo.; f Reproductive and Women’s Health, Intercountry support team World Health Organization, Ouagadougou, Burkina Faso.; g Department of Reproductive Health and Research, World Health Organization, Avenue Appia 20, 1211 Geneva 27, Switzerland.

## Abstract

**Problem:**

In Burkina Faso, the coverage of services for family planning is low due to shortage of qualified health staff and limited access to services.

**Approach:**

Following the launch of the Ouagadougou Partnership, an alliance to catalyse the expansion of family planning services, the health ministry created a consortium of family planning stakeholders in 2011. The consortium adopted a collaborative framework to implement a pilot project for task sharing in family planning at community and primary health-care centre levels in two rural districts. Stakeholders were responsible for their areas of expertise. These areas included advocacy; monitoring and evaluation; and capacity development of community health workers (CHWs) to offer oral and injectable contraceptives to new users and of auxiliary nurses and auxiliary midwives to provide implants and intrauterine devices. The health ministry implemented supportive supervision cascades involving relevant planning and service levels.

**Local setting:**

In Burkina Faso, only 15% (2563/17 087) of married women used modern contraceptives in 2010.

**Relevant changes:**

Adoption of new policies and clinical care standards expanded task sharing roles in family planning. The consortium trained a total of 79 CHWs and 124 auxiliary nurses and midwives. Between January 2017 and December 2018, CHWs provided injectables to 3698 new users, and auxiliary nurses or midwives provided 726 intrauterine devices and 2574 implants to new users. No safety issues were reported.

**Lessons learnt:**

The pilot project was feasible and safe, however, financial constraints are hindering scale-up efforts. Supportive supervision cascades were critical in ensuring success.

## Résumé

**Problème:**

Au Burkina Faso, la couverture des services de planification familiale est faible, en raison de la pénurie de personnel de santé qualifié et de l'accès limité à ces services.

**Approche:**

Suite au lancement du Partenariat de Ouagadougou, alliance destinée à accélérer l'expansion des services de planification familiale, le ministère de la Santé a créé en 2011 un consortium d'acteurs de la planification familiale. Ce consortium a adopté un cadre collaboratif pour mettre en œuvre un projet pilote visant à partager les tâches de planification familiale, aux niveaux de la communauté et des centres de soins primaires, dans deux districts ruraux. Les différents acteurs étaient responsables de leurs domaines d'expertise, parmi lesquels la sensibilisation, le suivi et l'évaluation, ainsi que le renforcement des compétences des agents de santé communautaires, afin de proposer des contraceptifs oraux et injectables à de nouvelles utilisatrices, et des aides-soignants et sages-femmes auxiliaires, dans le but de poser des implants et des dispositifs intra-utérins. Le ministère de la Santé a mis en place des structures d'encadrement faisant intervenir les niveaux concernés de planification et de services.

**Environnement local:**

Au Burkina Faso, seules 15% (2563/17 087) des femmes mariées utilisaient des contraceptifs modernes en 2010.

**Changements significatifs:**

L'adoption de nouvelles politiques et de nouveaux standards de soins a élargi les rôles de partage des tâches en matière de planification familiale. Le consortium a formé un total de 79 agents de santé communautaires et de 124 aides-soignants et sages-femmes auxiliaires. Entre janvier 2017 et décembre 2018, les agents de santé communautaires ont délivré des contraceptifs injectables à 3698 nouvelles utilisatrices, et les aides-soignants et sages-femmes auxiliaires ont posé 726 dispositifs intra-utérins et 2574 implants à de nouvelles utilisatrices. Aucun problème de sécurité n'a été signalé.

**Leçons tirées:**

Ce projet pilote était réalisable et sûr; cependant, des contraintes financières entravent les efforts d'évolution. Les structures d'encadrement ont été essentielles à la réussite de ce projet.

## Resumen

**Situación:**

En Burkina Faso, la cobertura de los servicios de planificación familiar es baja debido a la escasez de personal sanitario cualificado y al acceso limitado a los servicios.

**Enfoque:**

Tras la puesta en marcha de Ouagadougou Partnership (la Asociación de Uagadugú), una alianza para promover la expansión de los servicios de planificación familiar, el Ministerio de Salud creó en 2011 un consorcio de interesados en la planificación familiar. El consorcio adoptó un marco de colaboración para ejecutar un proyecto piloto de distribución de tareas en materia de planificación familiar a nivel comunitario y de centros de atención primaria de la salud en dos distritos rurales. Las partes interesadas eran responsables de sus áreas de especialización. Estas áreas incluían la promoción, la vigilancia y la evaluación, y el desarrollo de la capacidad de los trabajadores sanitarios de la comunidad (community health workers, CHW) para ofrecer anticonceptivos orales e inyectables a los nuevos usuarios, así como de los enfermeros auxiliares y las parteras auxiliares para proporcionar implantes y dispositivos intrauterinos. El Ministerio de Salud implementó cascadas de supervisión de apoyo que involucran niveles relevantes de planificación y servicio.

**Marco regional:**

En Burkina Faso, solamente el 15 % (2 563/17 087) de las mujeres casadas utilizaron anticonceptivos modernos en 2010.

**Cambios importantes:**

La adopción de nuevas políticas y estándares de atención clínica expandió la distribución de tareas en la planificación familiar. El consorcio capacitó a un total de 79 CHW y 124 enfermeros y parteras auxiliares. Entre enero de 2017 y diciembre de 2018, los CHW administraron inyectables a 3 698 nuevas usuarias, y los enfermeros auxiliares o parteras suministraron 726 dispositivos intrauterinos y 2 574 implantes a las nuevas usuarias. No se informó de ningún problema de seguridad.

**Lecciones aprendidas:**

El proyecto piloto era viable y seguro; sin embargo, las limitaciones financieras están obstaculizando los esfuerzos de ampliación. Las cascadas de supervisión de apoyo fueron fundamentales para garantizar el éxito.

## ملخص

**المشكلة:**

انخفاض تغطية خدمات تنظيم الأسرة بسبب نقص العاملين الصحيين المؤهلين ومحدودية الوصول إلى الخدمات.

**الأسلوب:**

عقب إطلاق شراكة واغادوغو وهو تحالف لتحفيز توسع خدمات تنظيم الأسرة، شكلت وزارة الصحة ائتلافًا من أصحاب المصلحة في تنظيم الأسرة في عام 2011. اعتمد الائتلاف إطارًا تعاونيًا لتنفيذ مشروع تجريبي خاص بمشاركة المهام المتعلقة بتنظيم الأسرة على مستوى مراكز الرعاية الصحية الأولية والمجتمع في منطقتين ريفيتين. وكان أصحاب المصلحة مسؤولين عن مجالات خبراتهم. وشملت هذه المجالات: التأييد؛ والمراقبة والتقييم؛ وتطوير قدرات العاملين في مجال الصحة المجتمعية (CHW) لتقديم موانع الحمل التي يتم تعاطيها عن طريق الفم، والقابلة للحقن إلى مستخدمين جدد؛ بالإضافة إلى تطوير قدرات الممرضات المساعدات والقابلات المساعدات لتوفير أدوات زرع داخل الرحم وأجهزة رحمية لمنع الحمل. نفذت وزارة الصحة سلاسل إشراف داعمة تتضمن مستويات التخطيط والخدمات ذات الصلة.

**المواقع المحلية:**

استخدمت 15% فقط (2563/17087) من النساء المتزوجات في بوركينا فاسو وسائل منع حمل حديثة في عام 2010.

**التغيّرات ذات الصلة:**

أدى اعتماد سياسات جديدة ومعايير الرعاية السريرية إلى توسيع أدوار مشاركة المهام المتعلقة بتنظيم الأسرة. ودرب الائتلاف إجمالي 79 عاملاً في مجال الصحة المجتمعية (CHW)، و124 ممرضة مساعدة وقابلة مساعدة. في الفترة ما بين يناير/كانون ثاني 2017 وديسمبر/كانون أول 2018، قدم العاملون في مجال الصحة المجتمعية (CHW) وسائلَا قابلة للحقن إلى 3698 مستخدمًا جديدًا، وقدمت الممرضات المساعدات والقابلات المساعدات 726 جهازًا رحمياً و2574 أداة للزرع داخل الرحم للمستخدمين الجدد. ولم يتم الإبلاغ عن أي مشاكل متعلقة بالسلامة.

**الدروس المستفادة:**

كان المشروع التجريبي عمليًا وآمنًا، ومع ذلك، كانت القيود المالية تعيق جهود التوسع. كانت سلاسل الإشراف الداعمة حاسمة في ضمان النجاح.

## 摘要

**问题:**

在布吉纳法索，由于缺乏合格的医务人员以及获得服务的途径有限，计划生育服务的覆盖率很低。

**方法:**

在瓦加杜古伙伴关系（旨在促进推广计划生育服务的联盟）启动之后，卫生部于 2011 年成立了一个计划生育利益相关者联盟。该联盟采用协作架构以实施试点项目，从而在两个农村地区的社区和初级医疗保健中心实现计划生育任务分担。利益相关者对其专业领域负责。这些领域包括加大宣传、监测与评估，培养社区卫生工作者 (CHW) 向新用户提供口服型和注射型避孕药的能力，以及由助理护士和助理助产士提供植入型避孕措施和宫内节育器的能力。卫生部实施了支持性联动监督，涉及相关的计划和服务层面。

**当地状况:**

2010 年，布吉纳法索仅有 15% （即 2563/17087）的已婚妇女使用现代避孕措施。

**相关变化:**

新的政策和临床护理标准的采用扩大了任务分担在计划生育中的作用。联盟共培训了 79 名社区卫生工作者以及 124 名助理护士和助产士。2017 年 1 月至 2018 年 12 月间，社区卫生工作者向 3698 名新用户提供了注射型避孕药物，助理护士或助产士向新用户提供了 726 个宫内节育器和 2574 个植入型避孕措施。目前尚未出现任何安全问题报告。

**经验教训:**

试点项目可行且安全，但是，财政拮据影响了其规模的进一步扩大。支持性联动监督是成功的关键。

## Резюме

**Проблема:**

В Буркина-Фасо наблюдается низкий уровень охвата услугами по планированию семьи по причине нехватки квалифицированного медицинского персонала и ограниченного доступа к услугам.

**Подход:**

После запуска программы «Партнерство Уагадугу» (союза, в задачи которого входит ускорение расширения охвата услугами по планированию семьи) Министерство здравоохранения в 2011 году создало консорциум партнеров по данному вопросу. Консорциум определил рамки сотрудничества для осуществления пилотного проекта по разделению функций в оказании услуг по планированию семьи на уровне общин и центров первичного медико-санитарного обслуживания в двух сельских регионах. Каждый из партнеров отвечал за свою область знаний. Эти области знаний включали информационно-разъяснительную работу, мониторинг и оценку, а также обучение общинных медико-санитарных работников (ОМСР) тому, чтобы они могли предлагать пероральные и инъекционные контрацептивы новым пользователям, а также обучение младших медсестер и акушерок процедуре установки имплантатов и внутриматочных устройств. Министерство здравоохранения также внедрило ступенчатую систему надзора и поддержки, в которую вошли соответствующие уровни планирования и оказания услуг.

**Местные условия:**

В 2010 году только 15% замужних женщин в Буркина-Фасо (2563 из 17 087) использовали современные контрацептивы.

**Осуществленные перемены:**

Принятие новых правил и стандартов клинического ухода расширило возможности разделения функций в услугах планирования семьи. Консорциум обучил в общей сложности 79 ОМСР и 124 младшие медсестры и акушерки. В период с января 2017 года по декабрь 2018 года ОМСР выдали инъекционные контрацептивы 3698 новым пользователям, а младшие медсестры и акушерки установили 726 внутриматочных устройств и 2574 имплантата. Проблем с безопасностью при этом не было зарегистрировано.

**Выводы:**

Пилотный проект оказался осуществимым и безопасным, однако финансовые ограничения препятствуют расширению его масштабов. Критическим звеном в обеспечении успешности проекта оказалась ступенчатая система надзора и поддержки.****

## Introduction

Family planning is recognized as a cost–effective intervention for curbing issues within sexual and reproductive health, improving women’s status and increasing women’s capacities to contribute to family income.^1^^,^^2^ Yet, access to and uptake of family planning services in sub-Saharan Africa remain low,^3^^,^^4^ resulting in many unintended pregnancies. In Western Africa, between 2010 and 2015, this low service coverage has resulted in a fertility rate as high as 5.5 children per woman and poor maternal and child outcomes.^5^ To address the situation and catalyse the expansion of family planning services, nine francophone African countries, including Benin, Burkina Faso, Côte d’Ivoire, Guinea, Mali, Mauritania, Niger, Senegal and Togo, launched the Ouagadougou Partnership in 2011.

The common challenges of low service coverage for family planning and shortage of qualified human resources prompted the partnership to identify task sharing as a promising intervention to reduce the high levels of unmet need in family planning.^6^^,^^7^ All partnership countries included task sharing for family planning into their priority commitments and defined strategic steps to boost policy changes and field implementation.

This article reports key lessons from Burkina Faso. We used the World Health Organization’s (WHO’s) *A guide to identifying and documenting best practices in family planning programmes*^8^ to document task sharing activities, drawing from reports, meeting notes and proceedings, project data, and interviews with key stakeholders at national and regional levels.

## Local setting

In Burkina Faso, only 15% (2563/17 087) of married women used modern contraceptives in 2010, with 20-percentage points difference between urban (31%) and rural areas (11%). The unmet needs for family planning were estimated at 24% (4069/17 087) with also a rural versus urban gap.^9^ Expanding coverage of family planning services was therefore needed, particularly in rural settings, where major challenges include geographical access to health facilities, and predominantly low- and middle-level qualified health-care providers. For example, in 2015, national statistics showed that 48% (87/181) of non-physician staff working in the 39 primary health-care centres of the rural Tougan health district were auxiliary nurses and auxiliary midwives, while in the rural Dandé health district, this figure was 54% (66/122) for 29 primary health-care centres.^10^

Some form of task sharing in family planning have existed in the country since the Declaration of Alma Ata in 1978. At the community level, community health workers (CHWs) can counsel clients, distribute condoms and resupply women with oral contraceptives. At the primary health-care centre level, auxiliary nurses or auxiliary midwives can provide short-acting contraceptive methods, including injectable contraceptives. However, health policies and clinical care standards prohibited them to perform more complex tasks. CHWs could not prescribe oral contraceptive pills to new users and provide injectable contraceptives, and auxiliary nurses or midwives could not insert and remove implants or intrauterine devices.

## Approach

Following the Ouagadougou Partnership launch in 2011, the health ministry created a consortium of family planning stakeholders comprising nongovernmental organizations (NGOs), including *Association Burkinabé pour le Bien-Être Familial*, *Équilibre et Populations* and Marie Stopes International. Partners agreed on a framework centred on collaboration and committed to specific responsibilities in their respective areas of expertise, such as advocacy, capacity development of CHWs, auxiliary nurses and auxiliary midwives and monitoring and evaluation.

Between 2017 and 2018, the consortium conducted a pilot project for task sharing in family planning in Tougan and Dandé, two rural districts located in different regions of the country. A total of 25 primary health-care centres and their catchment areas were included in both districts. The strategic approach encompassed an advocacy strategy for changes in policies and clinical care standards; community outreach activities to increase the demand for services; and staff capacity strengthening at both community and primary health-care centre levels. All the activities were conducted in close collaboration with the district level health ministry. 

### Policy and standard changes

To change health policies and clinical care standards for supporting task sharing in family planning, the consortium developed an advocacy strategy in 2012, which was based on the regular review of scientific evidence, including data from the field, during consortium meetings. The consortium established a steering committee to oversee the entire project. In addition to senior representatives of the health ministry and implementing NGOs, this committee comprised key partners at country level, including WHO, the United Nations Population Fund and the United States Agency for International Development. The consortium shared evidence on task sharing in family planning, through oral presentations and written summaries with the committee and during meetings with authorities at the provincial, regional and national levels before the project started as well as providing new results on the project’s feasibility, effectiveness and patient safety during the project.

### Demand creation activities

*Association Burkinabé pour le Bien-Être Familial* was responsible for demand creation activities by training CHWs to conduct more home visits and discussions with increased focus on family planning at the community level. CHWs learnt counselling and the delivery of key messages during the capacity strengthening workshops. CHWs visited community events, such as market days, to deliver the key messages to relevant audience. Demand creation was also facilitated by strengthening the capacity of auxiliary nurses and midwives for quality counselling at facility level, which was included in their capacity strengthening workshops.

### Capacity strengthening

#### CHWs

With the support of the other members of the consortium, *Association Burkinabé pour le Bien-Être Familial* led the training of CHWs. These providers were already working in different programmes, such as home management of malaria or undernutrition screening and had been recruited by the health ministry. Their monthly incentive approximates 40 United States dollars and they did not receive additional payment for the additional tasking sharing. The association organized two sessions in Dandé and three in Tougan. During the two-week workshops, for which the participants had to leave work, they received training on family planning counselling and the provision of condoms and oral and injectable contraceptives. The training method included interactive presentations, group work, role-play and practical sessions in health facilities.

#### Auxiliary nurses and midwives

*Marie Stopes International* facilitated the training of auxiliary nurses and midwives on implant and intrauterine device counselling, insertion, and removal. Two workshops were organized in Dandé and three in Tougan. Each workshop lasted two weeks, had full training days and used interactive presentations, group works and practical sessions.

### Monitoring and evaluation 

*Équilibre et Populations* coordinated the implementation of the agreed monitoring and evaluation plan, which included monitoring service provision issues on both client and provider sides, reviewing periodic and final project reports, and ensuring the implementation of recommendations.

### Supportive supervision

**Fig. 1:**
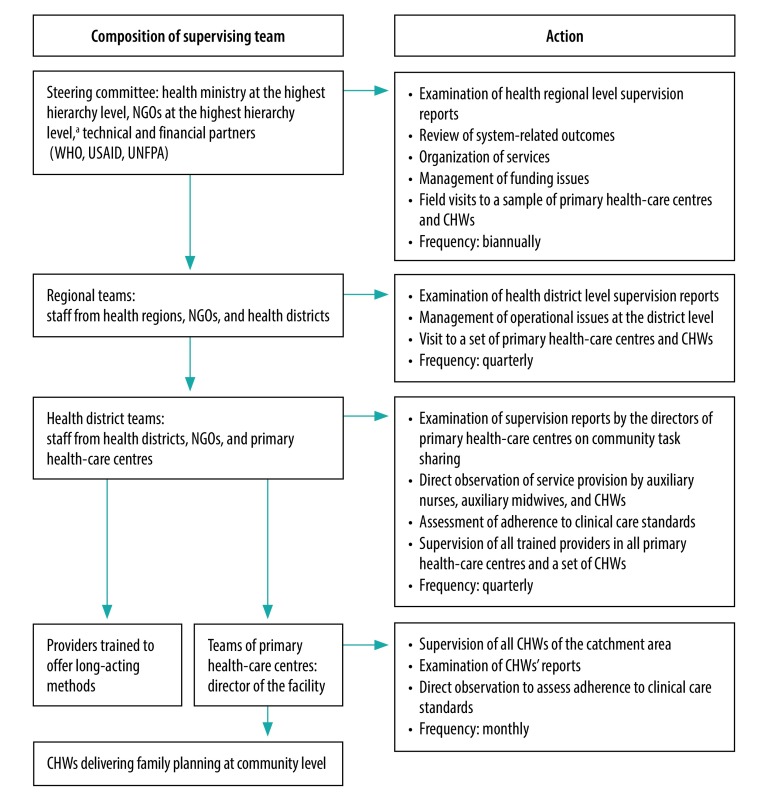
Supervision cascade related to task sharing in family planning, Burkina Faso

CHW: community health worker; NGO: nongovernmental organization; UNFPA: United Nations Population Fund; USAID: United States Agency for International Development; WHO: World Health Organization.

^a^ NGOs included were *Association Burkinabé pour le Bien-Être Familial* and Marie Stopes International.

The health ministry and NGOs created a supportive supervision strategy, cascading from the national level to the community and primary health-care centre levels (Fig. 1). This cascade strengthened the management system at the health district and regional levels, as well as the service provision activities at the level of primary health-care centres and CHWs. The health district team supervised the activities of the trained auxiliary nurses and midwives quarterly and the director of each primary health-care centre supervised the work of CHWs monthly. Each director was also engaged in the supervising health district team as to ensure the follow-up of the recommendations of the health district team. The regional team supervised the activities at the district level, and a national steering committee oversaw the entire process.

## Relevant changes

The advocacy process raised awareness of relevant actors, which contributed to the adoption of new policies and clinical care standards allowing CHWs to prescribe oral contraceptives and provide injectable contraceptives to new users at the community level and auxiliary nurses and midwives to offer implants and intrauterine devices at primary health-care centres.

Between January 2017 and December 2018, the consortium trained a total of 79 CHWs and 124 auxiliary nurses and midwives and implemented the pilot project that resulted in an increase in the numbers of new family planning users in both districts. From February 2017 to December 2018, CHWs provided injectables to 3698 new contraceptive users and auxiliary nurses or midwives provided 726 new intrauterine devices and 2574 new implants (Table 1). During the project period, the uptake of family planning services from trained providers remained relatively steady at both community and primary health-care centre levels, and no safety incidents were reported. CHWs were able to identify and manage contraceptive side-effects, and to refer clients to primary health-care centres if needed. In 2018, CHWs referred 60 contraceptive users for further management of side-effects.

**Table 1:** Provision of contraceptives to new users by provider, Burkina Faso, 2017–2018

Period	Dandé district						Tougan district				
	CHWs			Auxiliary staff^a^			CHWs			Auxiliary staff^a^	
	No. of injectables	No. of pills		No. of intrauterine devices	No. of implants		No. of injectables	No. of pills		No. of intrauterine devices	No. of implants
2017-Q1	20	1		12	61		0	0		0	0
2017-Q2	178	45		91	303		354	30		45	279
2017-Q3	162	62		58	127		291	91		32	84
2017-Q4	235	77		41	163		417	93		115	228
2018-Q1	168	53		41	207		341	85		78	122
2018-Q2	239	71		38	262		390	98		55	221
2018-Q3	143	41		22	76		246	54		36	100
2018-Q4	208	71		22	152		306	67		40	189
**Total**	**1353**	**421**		**325**	**1351**		**2345**	**518**		**401**	**1223**

CHW: community health worker; Q: quarter.

^a^ Includes auxiliary nurses and midwives.

## Lessons learnt

Gathering different family planning stakeholders within the same country to plan and act collaboratively contributed to increasing access for women and couples to a wider range of contraceptive methods and to meeting the demand of women and couples for family planning.

The iterative evidence-based advocacy efforts proved useful in changing policies and clinical care standards. The pilot project reinforced the existing evidence^6^ that injectables can be safely administered by CHWs and implants and intrauterine devices by auxiliary nurses and midwives. The supportive supervision approach, combined with the engagement of the steering committee, was considered critical to ensure the success of the project as the supervision allowed a continuous and dynamic process of contribution, enrichment and nurturing among all consortium stakeholders, including the health ministry (Box 1).

Box 1 **Summary of main lessons learnt**

• Task sharing in family planning was feasible and safe at both community and primary health-care centre levels.

• Mobilizing key stakeholders catalysed changes in health policies and clinical care standards related to task sharing in family planning.

• Participatory supervision cascades under the leadership of the health ministry was critical in ensuring the success of the project.

There were challenges to the task sharing project. First, the high turnover of auxiliary nurses and midwives in the project areas was an issue, which the consortium addressed by offering training sessions to newly arrived auxiliary nurses and midwives. This issue will likely have less impact once this task sharing is implemented in the whole country. Second, the long-term sustainability of task sharing in family planning at the community level requires further study, as CHWs are not yet fully integrated into the formal health system and limited salary incentives affect their motivation. Third, primary health-care providers equipped with additional competencies may legitimately wish for a better salary. Fourth, following the overall positive processes and outcomes of the pilot project,^11^ stakeholders agreed to scale up the project nationwide in 2018. Although financial constraints are delaying the scale up, the country adopted a scaling-up plan for task sharing in family planning in late 2018, for which funding for its operationalization needs to be identified.

Countries facing similar challenges in coverage of family planning services could implement an equivalent strategy so that women and couples can have access to a wider range of safe contraceptive services that better meet their needs.
